# Cardiac hydatidosis mimicking ischemic heart disease: A case report

**DOI:** 10.1016/j.idcr.2026.e02512

**Published:** 2026-02-10

**Authors:** Andres Chaponan-Lavalle, Cherie Quiroz Cortegana, Luis Ivan Gordillo Velasquez, Jaime Caceres Pizarro, Nelson Diaz, Jorge Alave

**Affiliations:** a Universidad Peruana de Ciencias Aplicadas, Lima, Peru; b Clinica Good Hope, Lima, Peru; c Hospital Cayetano Heredia, Lima, Peru; d Universidad Peruana Unión, Lima, Peru

**Keywords:** Echinococcosis, *Echinococcus granulosus*, Cysts

## Abstract

Cystic echinococcosis (CE), caused by *Echinococcus granulosus*, is a zoonotic disease with cardiac involvement reported in less than 2 % of cases but associated with significant clinical challenges. We describe a 44-year-old woman from Peru who presented with exertional chest pain and T-wave inversions on electrocardiography. Imaging revealed a large multiloculated cystic mass with peripheral calcifications in the left ventricular wall, and Western Blot serology confirmed CE. The patient underwent surgical excision under cardiopulmonary bypass, followed by albendazole therapy. Histopathology confirmed a non-viable hydatid cyst. Postoperative recovery was uneventful, and she remained asymptomatic at 16-month follow-up. This case underscores the importance of considering cardiac hydatidosis in endemic regions when evaluating patients with angina-like symptoms in the presence of a cystic cardiac mass on imaging. Public awareness, improved medical education, and ongoing research are crucial to better managing this condition.

## Introduction

Cystic echinococcosis (CE) is a zoonotic disease caused by *Echinococcus granulosus* sensu lato [1]. In humans, who serve as accidental intermediate hosts, the parasite’s eggs are ingested via contaminated food or water. Oncospheres released in the intestine penetrate the mucosa and disseminate hematogenously [2].

Hydatidosis is a significant public health problem in South America, with over 45,000 cases reported between 2009 and 2018 in the Americas. Peru has the highest incidence, with annual rates ranging from 7 to 11 cases per 100,000 populations [3]. Hydatid cysts most commonly affect the liver (55–70 %) and the lungs (18–35 %), with simultaneous involvement in 5–13 % of cases, while cardiac CE represents 2 % of cases [4].

We report a case of a 44-year-old woman from Junín, Peru, who experienced angina pectoris and an abnormal electrocardiogram (ECG) pattern suggestive of coronary artery disease.

## Case history

A 44-year-old woman from the Peruvian highlands presented with progressive exertional chest pain for six months, accompanied by neck stiffness. She had a history of chronic hyperthyroidism treated with methimazole and a pulmonary hydatid cyst resection 15 years prior. On examination, she was hemodynamically stable, with no murmurs.

**Fig. 1:**
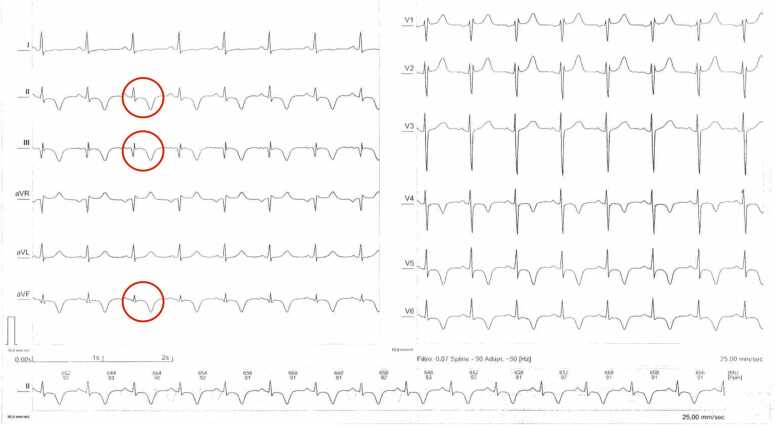
ECG showing sinus rhythm (HR: 90 bpm), negative T waves in leads II, III, aVF, V4–V6 suggestive of subepicardial ischemia, and RR' in V1–V2 indicative of incomplete right bundle branch block.

**Fig. 2:**
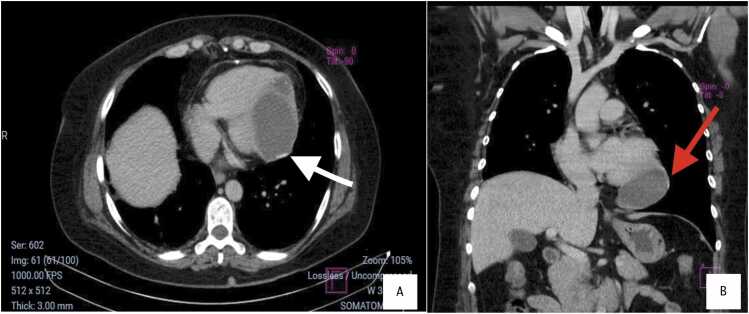
(A) Axial chest CT image showing a multiloculated hypodense mass within the apical region of the left ventricle, with peripheral contrast enhancement and calcifications (95 mm × 47 mm × 68 mm) (white arrow).(B) Coronal reconstruction revealing the full extent of the lesion, with peripheral calcifications (red arrows) and no involvement of the mediastinum.

Laboratory studies showed a white blood cell count of 7200/mm^3^ (reference range: 4000–11,000/mm³) with 2.1 % eosinophils (reference range: 0–6 %), and a troponin I level of 0.01 ng/mL (reference range: <0.04 ng/mL). ECG demonstrated T-wave inversions in leads II, III, aVF, and V4–V6 (Fig. 1). Transthoracic echocardiography revealed a hyperechoic mass in the apical region of the left ventricle with preserved systolic function. A contrast-enhanced computed tomography (CT) scan of the chest showed a 95 × 47 × 68 mm multiloculated hypodense cystic mass with peripheral calcifications in the left ventricular wall (Fig. 2). Coronary angiography ruled out obstructive coronary artery disease. Western Blot serology confirmed the diagnosis of cystic echinococcosis.

**Fig. 3:**
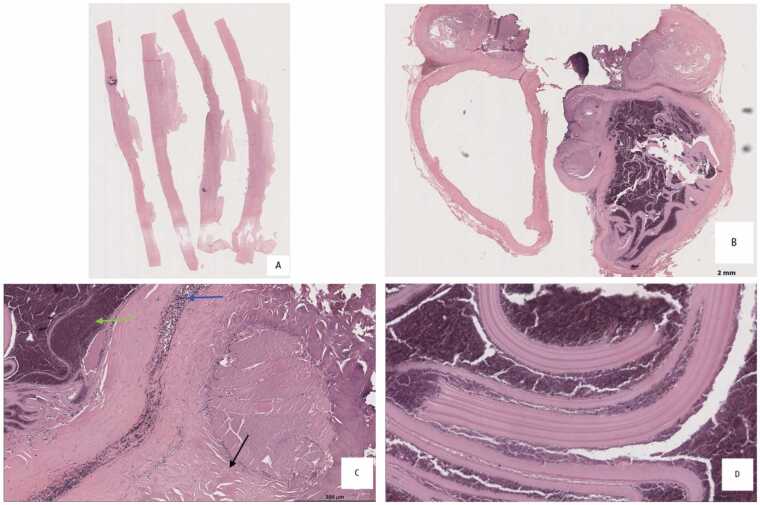
Hematoxylin and eosin–stained sections from a surgically resected cardiac hydatid cyst. (A) Fibrosclerotic cyst wall with focal calcification membrane (B) Fibrous (adventitial) wall sections delineating hydatid cyst membrane. (C) Fibrosclerotic wall with chronic inflammatory infiltrate (blue arrow), associated with degenerated hydatid membranes and cellular debris (green arrow); adjacent striated muscle fibers are also identified (black arrow). (D) Lamelar, acellular tissue consistent with hydatid cyst membranes.

Surgical resection via median sternotomy and cardiopulmonary bypass was performed. After isolating the field with hypertonic saline-soaked gauze, the cyst was incised, revealing yellow fluid, laminated membranes, and necrotic material. The affected myocardial area was resected and repaired with a pericardial patch. Tissue was sent for histopathological analysis (Fig. 3). Postoperative recovery was uneventful and albendazole 800 mg daily for 15 days was administered. Sixteen months later, she experienced dizziness and bradycardia (heart rate: 52 bpm). Although ECG showed repolarization abnormalities, stress tests and Holter monitoring ruled out ischemia or arrhythmias, indicating an adequate chronotropic response and New York Heart Association (NYHA) Functional Class I.

## Discussion

Cardiac hydatid involvement occurs in less than 2 % of CE cases and typically results from hematogenous spread via the coronary arteries. Cardiac hydatidosis most frequently affects the left ventricle (34 %) due to its rich blood supply, followed by the interventricular septum and right ventricle [2], [5]. Growth is usually slow and asymptomatic until cysts enlarge enough to cause compression of adjacent structures. Presentations range from chest pain, arrhythmias, syncope, or conduction abnormalities to tamponade or embolic events in case of rupture [6]. Angina-like pain is rare and typically reflects local mass effect, as in our patient [5].

Imaging plays a central role in diagnosis. Echocardiography may show well-defined cystic lesions with or without internal septations. CT scan provides detailed information on cyst morphology, location, calcifications, and relation to surrounding structures. In our patient, CT scan showed a large multiloculated cyst with peripheral calcifications, a typical pattern (Fig. 2). Cardiac Magnetic Resonance Imaging (MRI) may offer additional detail, especially in evaluating wall involvement or complications [7]. Definitive diagnosis is confirmed by histopathology, as in this case, which showed laminated *acellular* membranes and fibrous connective tissue without protoscolex, consistent with a non-viable hydatid cyst (Fig. 3).

Serologic testing can support diagnosis, though sensitivity varies with cyst location. Immunoblot assays are currently preferred due to greater specificity compared to Enzyme-linked immunosorbent assay (ELISA) or Indirect hemagglutination assay (IHA), especially for extrahepatic disease [8]. In our case, the Western Blot serology confirmed the diagnosis. Eosinophilia occurs in 40 % of CE cases, often linked to cyst rupture or leakage [9]. Its absence here likely reflects a contained lesion.

Treatment of cardiac hydatid cysts generally requires surgical excision, irrespective of cyst stage, given the risk of rupture or embolization. This contrasts with hepatic CE, where World Health Organization (WHO) classification allows medical therapy or observation in inactive CE cases (CE4, CE5). Surgical strategies depend on cyst location [1]. Off-pump surgery may suffice for superficial lesions, whereas deeper myocardial cysts, like in this case, necessitate cardiopulmonary bypass [1], [5]. Use of scolicidal agents such as hypertonic saline is essential to reduce recurrence or anaphylaxis [1], [2].

Albendazole remains the first-line antiparasitic agent due to its superior tissue penetration and efficacy [1]. It is typically administered pre- and postoperatively, although data on optimal duration for cardiac cases are limited. The 2010 WHO consensus recommends initiating therapy at least one day before surgery and continuing for a minimum of one month postoperatively [1], [5].

The prognosis of cardiac CE after complete resection is generally favorable. Reported mortality has declined significantly with early diagnosis and surgical intervention [1]. Regular postoperative imaging is advised to monitor for recurrence [1], [10]. In this patient, follow-up at 16 months showed no evidence of cyst recurrence, and functional status remained preserved.

## Conclusion

Cardiac hydatid disease is a rare condition with significant public health relevance in South America. Cystic echinococcosis should be considered in endemic regions when evaluating patients with angina-like symptoms and a cystic cardiac mass on imaging. Early recognition, increased public awareness, and ongoing research are essential to improve management and prevent potentially serious complications.

## CRediT authorship contribution statement

**Quiroz-Cortegana Cherie:** Writing – original draft, Validation, Investigation, Formal analysis, Data curation. **Andres Chaponan-Lavalle:** Writing – review & editing, Writing – original draft, Validation, Supervision, Project administration, Investigation. **Jaime Caceres Pizarro:** Writing – original draft, Validation, Supervision. **Luis Ivan Gordillo Velasquez:** Investigation, Formal analysis, Data curation. **Jorge Alave:** Writing – review & editing, Writing – original draft, Validation, Supervision, Project administration, Investigation, Formal analysis, Data curation. **Nelson Diaz:** Writing – review & editing, Writing – original draft, Validation, Investigation, Formal analysis, Data curation.

## Declaration of Competing Interest

The authors declare that they have no known competing financial interests or personal relationships that could have appeared to influence the work reported in this paper.

## References

[1] Banisefid E., Baghernezhad K., Beheshti R., Hamzehzadeh S., Nemati S., Samadifar Z. (2023). Cardiac hydatid disease; a systematic review. BMC Infect Dis [Internet].

[2] Brunetti E., Kern P., Vuitton D.A. (2010). Writing Panel for the WHO-IWGE. Expert consensus for the diagnosis and treatment of cystic and alveolar echinococcosis in humans. Acta Trop.

[3] Hidatidosis / Equinococosis - OPS/OMS | Organización Panamericana de la Salud [Internet]. [cited 2023 Jul 15]. Available from: 〈https://www.paho.org/es/temas/hidatidosis-equinococosis〉.

[4] Goyal S., Goyal S., Sangwan S., Sachar S. (2014). Uncommon locations and presentations of hydatid cyst. Ann Med Health Sci Res [Internet].

[5] Bumann S., Kuenzli E., Lissandrin R., Brunetti E., Goblirsch S., Henning L. (2024). Cardiac cystic echinococcosis—a systematic review and analysis of the literature. Casulli A., editor. PLoS Negl Trop Dis [Internet].

[6] Shojaei E., Yassin Z., Rezahosseini O. (2016). Cardiac hydatid cyst: a case report. Iran J Public Health.

[7] Durhan G., Tan A.A., Düzgün S.A., Akkaya S., Arıyürek O.M. (2020). Radiological manifestations of thoracic hydatid cysts: pulmonary and extrapulmonary findings. Insights Imaging [Internet].

[8] Wen H., Vuitton L., Tuxun T., Li J., Vuitton D.A., Zhang W. (2019). Echinococcosis: advances in the 21st century. Clin Microbiol Rev [Internet].

[9] Collado-Aliaga J., Romero-Alegría Á., Alonso-Sardón M., López-Bernus A., Galindo-Pérez I., Muro A. (2019). Eosinophilia and cystic echinococcosis: what is the relationship?. Trans R Soc Trop Med Hyg [Internet].

[10] Brunetti E., White A.C. (2012). Cestode infestations: hydatid disease and cysticercosis. Infect Dis Clin North Am.

